# O21 - The role of DNA damage and repair in allergic airway inflammation

**DOI:** 10.1186/2045-7022-4-S1-O21

**Published:** 2014-03-14

**Authors:** Tze Khee Chan, Xin Yi Loh, Daniel WS Tan, Bevin P Engelward, Fred WS Wong

**Affiliations:** 1 Department of Pharmacology, Yong Loo Lin School of Medicine, National University Health System, Singapore; 2 Immunology Program, Life Science Institute, National University of Singapore, Singapore; 3 Interdisciplinary Research Group in Infectious Diseases, Singapore-MIT Alliance for Research and Technology (SMART), Singapore; 4 Department of Biological Engineering, Massachusetts Institute of Technology, Cambridge, MA, USA

## Introduction

Extensive DNA damage and inefficient DNA repair might be responsible for some of the pathogenic features in patients suffering from asthma. To determine whether DNA adducts can be used as a “dosimeter” for asthma disease severity, we measured the DNA adducts level in lung of mouse with house dust mite (HDM)-induced allergic airway inflammation, as the disease progresses. Apoptosis of airway epithelial cells is one of the most critical pathophysiological factors in the development of chronic asthma. As repairing of DNA lesions is important in preventing apoptosis, we propose that DNA repair plays an important pathophysiologic role in regulating lung epithelial cell DNA damage response.

## Results

We immunofluorescence-stained mice asthmatic lung tissue sections and observed an increase in DNA double strand break (DBS) markers, γH2AX and 53BP1 as compared to control. Level of DNA repair proteins that involved in homologous recombination and non-homologous end joining, were up-regulated substantially as early as 1 day-post last challenge. TUNEL assay revealed high level of DNA strand breaks in bronchial epithelium. DNA damage signaling pathway PCR array showed a reproducible increase in expression of multiple genes involved in DNA damage and repair. Treatment with glucocorticoid significantly reduced cell infiltration into airway as well as DNA damage and repair markers. To elucidate the role of DNA repair in regulating disease outcome, we treated mice with NU7441, a DNA-dependent protein kinases inhibitor, and had observed an increased DNA damage makers expression in lung and increased apoptotic level in bronchial epithelium as compare to no-drug treatment control. When DNA repair was obstructed, apoptosis of airway epithelium cells was enhanced. This indicates the important role of DNA repair in airway inflammation.

